# Unlocking the Cu-Co Interplay: Electrodeposited Spinel Co_2_CuO_4_ as a High-Performance Hydrogen Evolution Catalyst

**DOI:** 10.3390/ijms262211226

**Published:** 2025-11-20

**Authors:** Sankar Sekar, M. Mujtaba Momin, Abu Saad Ansari, Sangeun Cho, Youngmin Lee, Sejoon Lee, Abu Talha Aqueel Ahmed

**Affiliations:** 1 Division of System Semiconductor, Dongguk University-Seoul, Seoul 04620, Republic of Korea; sanssekar@dongguk.edu (S.S.); sangeun.c@dongguk.edu (S.C.); ymlee@dongguk.edu (Y.L.); sejoon@dongguk.edu (S.L.); 2 Quantum-Functional Semiconductor Research Center, Dongguk University-Seoul, Seoul 04620, Republic of Korea; 3 Department of Physics, Maharaja Sayajirao Gaikwad Arts, Science and Commerce College, Malegaon-Camp, Malegaon 423203, India; mohammedmujtaba1318@gmail.com; 4 Nano Center Indonesia Research Institute, Puspiptek Street, South Tangerang 15314, Banten, Indonesia; saad@nano.or.id

**Keywords:** Co_2_CuO_4_, Co_3_O_4_, electrodeposition, electrocatalysts, turnover frequency, hydrogen evolution reaction

## Abstract

Developing cost-effective and durable electrocatalysts with high hydrogen evolution efficiency remains a critical challenge for sustainable energy conversion. Herein, spinel-type Co_2_CuO_4_ and Co_3_O_4_ nanosheet electrodes were fabricated directly on Ni foam via a simple electrodeposition route and evaluated for the alkaline hydrogen evolution reaction (HER) in 1.0 M KOH. Structural and surface analyses confirmed the formation of phase-pure, porous, and highly interconnected nanosheet architectures, where the substitution of Cu^2+^ into the Co_3_O_4_ lattice induced charge-redistribution and optimized the electronic configuration. The Co_2_CuO_4_ catalyst exhibited superior activity, requiring an overpotential of 127 mV to achieve 10 mA cm^−2^ with a corresponding Tafel slope of 61 mV dec^−1^, outperforming the Co_3_O_4_ catalyst (176 mV and 95 mV dec^−1^). This enhancement arises from improved intrinsic kinetics, higher turnover frequency, and reduced charge-transfer resistance, reflecting an increased density of active sites and enhanced interfacial conductivity. Furthermore, the Co_2_CuO_4_ catalyst maintained excellent stability for 100 h at both 10 and 500 mA cm^−2^, attributed to its strong adhesion and open nanosheet framework, which facilitates efficient gas release and electrolyte diffusion. These findings establish Co_2_CuO_4_ as a promising and durable HER electrocatalyst for alkaline water electrolysis.

## 1. Introduction

The global pursuit of carbon-neutral energy systems has accelerated the development of sustainable and efficient hydrogen production technologies [1,2,3]. Among various strategies, water electrolysis stands out as a clean and scalable route to generating high-purity hydrogen [4,5,6]. However, the overall efficiency of water splitting is largely limited by sluggish electrode kinetics, particularly during the oxygen evolution reaction under alkaline conditions [7,8]. Although Pt-based catalysts are the benchmark for the hydrogen evolution reaction (HER) owing to the near-zero overpotential and rapid kinetics, their scarcity and high cost hinder their use in practical, large-scale applications [9]. Therefore, intensive research efforts have been devoted to exploring earth-abundant transition-metal oxides as cost-effective HER electrocatalyst alternatives to noble-metal catalysts. Among these materials, spinel-type Co_3_O_4_ has gained significant attention due to its rich redox chemistry (Co^2+^/Co^3+^), structural stability, and tunable electronic properties [10,11,12,13,14]. However, the intrinsically poor electrical conductivity and limited exposure of catalytically active sites hinder rapid charge-transfer and proton reduction, leading to moderate HER performance [15]. To address these challenges, cationic substitution has emerged as an effective strategy to modulate the electronic structure, optimize active-site distribution, and enhance catalytic activity [16].

In particular, incorporating Cu^2+^ into the Co_3_O_4_ lattice forms Co_2_CuO_4_, a bimetallic spinel oxide that enhances electron delocalization, promotes multiple redox transitions (Cu^+^/Cu^2+^ and Co^2+^/Co^3+^), and facilitates efficient charge-transport across the catalyst-electrolyte interface [14,17,18]. In addition to compositional optimization, morphological engineering is a key determinant of electrochemical performance, as the catalytic HER activity of transition-metal oxides depends strongly on their shape, size, and structural dimensionality [19,20]. Controlling these features allows the modulation of surface atom exposure, defect density, and charge-migration pathways. Among the various morphologies explored, such as nanoparticles, bulk materials, and nanocubes, two-dimensional (2D) interconnected nanosheet networks have demonstrated superior activity due to their high surface-to-volume ratio, short ion/electron diffusion lengths, and abundant unsaturated edge sites [20,21,22]. Their porous and mechanically robust framework, featuring an open nanosheet architecture, facilitates rapid electrolyte penetration and efficient gas release, thereby enhancing electrolyte accessibility and ensuring strong structural stability during prolonged electrolysis [23,24]. Recent studies have demonstrated that directly grown Co_2_CuO_4_ catalysts on Ni foam exhibit remarkable electrocatalytic activity. However, despite these advances, a systematic comparison of Co_2_CuO_4_ with its parent oxide for HER remains scarce, leaving an important gap in understanding how Cu incorporation modifies the electronic configuration and charge-transfer dynamics during hydrogen evolution.

In this study, we report a comparative investigation of Co_2_CuO_4_ and Co_3_O_4_ nanosheet catalysts synthesized directly on Ni foam via a simple electrodeposition approach. Comprehensive structural and electrochemical analyses reveal that the introduction of Cu^2+^ ions into the Co_3_O_4_ lattice modulates the cationic arrangement and electron density within the Co–O lattice, thereby enhancing the charge-transport through the lattice. The optimized Co_2_CuO_4_ catalyst exhibits an overpotential of 127 mV at a cathodic current density of 10 mA cm^−2^ with corresponding Tafel slope of 61 mV dec^−1^. The Co_2_CuO_4_ catalyst exhibit consistently smaller potential response compared to the pristine Co_3_O_4_ catalyst and even with Cu-Co-based catalyst (Table S1) when vigorously examined under various current densities and demonstrates excellent chronopotentiometric endurance even at a high current density of 500 mA cm^−2^. The TOF and ECSA analyses provide compelling evidence that Co_2_CuO_4_ possesses faster intrinsic reaction kinetics and superior site efficiency relative to Co_3_O_4_. The open nanosheet morphology, extensive interfacial contact with the Ni foam substrate, and well-connected conductive network collectively facilitate rapid electron/ion transport, accelerating the hydrogen evolution under alkaline conditions. This work uniquely bridges the existing gap through a comprehensive structural-electronic-electrochemical correlation, revealing how Cu substitution optimizes the spinel lattice and accelerates HER kinetics. The binder-free growth of nanosheet arrays on Ni foam offers a scalable and efficient electrode architecture. The findings not only establish a mechanistic understanding of Cu-induced electronic modulation but also propose a universal, design-guided approach for developing next-generation transition-metal oxide catalysts for sustainable hydrogen production.

## 2. Results

### 2.1. Morphological and Compositional Properties of Co_2_CuO_4_ and Co_3_O_4_ Films

The formation of Co_2_CuO_4_ and Co_3_O_4_ films on Ni foam follows a sequential electrochemical deposition and thermally induced transformation process. During the electrodeposition stage, the applied cathodic potential drives the reduction in metal salt ions and induces local water hydrolysis, generating the hydroxide ions near the substrate surface. These hydroxide ions react with Co^2+^ and Cu^2+^ ions in the electrolyte to form a mixed metal hydroxide intermediate molecule, which progressively nucleates and grows into nanosheet assemblies, as described in Equations (1) and (2):

Co^2+^ + Cu^2+^ + 4 (OH)^−^ → Co(OH)_2_↓ + Cu(OH)_2_↓, (1)

2 Co(OH)_2_ + Cu(OH)_2_ → Co_2_CuO_4_↓, (2)

**Figure 1:**
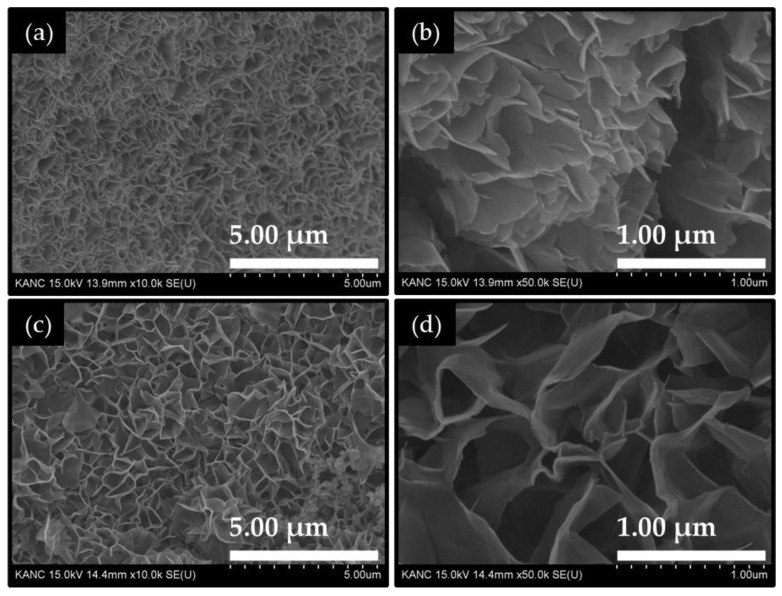
FE-SEM images of the electrodeposited (**a**,**b**) Co_3_O_4_ and (**c**,**d**) Co_2_CuO_4_ films recorded at different magnifications.

Figure 1 shows the field emission scanning electron microscope (FE-SEM) images of Co_2_CuO_4_ and Co_3_O_4_ nanosheet films. The pure Co_3_O_4_ film (Figure 1a) exhibits a dense array of ultrathin crumpled nanosheets, which uniformly cover the surface of the Ni foam substrate. These nanosheets are vertically aligned and interlinked, forming a three-dimensional porous network with open inter-sheet voids ranging from approximately 50 to 600 nm. This highly interconnected wrinkled nanosheet framework facilitates rapid electrolyte penetration and exposes a large number of electrochemically active edge sites, which are beneficial for catalytic reactions. The wrinkled sheet-like morphology indicates a high degree of structural flexibility and mechanical stability [25]. The smooth surface of the nanosheets (Figure 1b) and uniform coverage across the substrate suggest a controlled nucleation-growth balance during the electrodeposition process. The Co_2_CuO_4_ film (Figure 1c) demonstrates a distinctly evolved nanosheet structure. The thickness of these nanosheets ranges from approximately 17 to 35 nm, and more distinct voids are observed between these nanosheets (Figure 1d), exhibiting well-developed wrinkles and interconnected edges that create a 3D honeycomb-like architecture. The larger lateral growth of nanosheets and the wider inter-sheet voids between observed in the Co_2_CuO_4_ film compared to the Co_3_O_4_ film is a result of altered nucleation kinetics and the surface energy distribution during the electrodeposition. The inter-sheet spacing increases to several hundred nanometers, forming visible open channels that may serve as efficient pathways for electrolyte diffusion and gas bubble release during electrocatalytic HERs.

Thereafter, to confirm the elemental composition and spatial distribution of the nanosheet films, energy-dispersive X-ray spectroscopy (EDX) coupled with FE-SEM was employed for both Co_2_CuO_4_ and Co_3_O_4_ samples (Figure S1). The Co_2_CuO_4_ nanosheet film displays distinct EDX peaks corresponding to Co, Cu, and O constituents, validating the successful formation of the desired material phase. The quantitative analysis reveals an approximate atomic ratio of Co:Cu ≈ 2:1, which aligns closely with the expected stoichiometry of the spinel Co_2_CuO_4_ structure. The oxygen signal remains consistent with that of a well-oxidized phase, indicating complete conversion of the hydroxide precursor during annealing. Whereas the EDX spectrum of the Co_3_O_4_ nanosheet film reveals the prominent presence of Co and oxygen O peaks, verifying the successful formation of the binary cobalt oxide phase. The absence of any extraneous signals indicates the high purity of the films, as no residual precursor elements or substrate contamination were detected. The relatively high oxygen content corresponds well to the stoichiometric ratio of Co:O ≈ 3:4, consistent with the formation of the spinel Co_3_O_4_ phase.

### 2.2. Crystallographic and Bonding Properties of Co_2_CuO_4_ and Co_3_O_4_ Films

The structural characteristics of the electrodeposited Co_2_CuO_4_ and Co_3_O_4_ films were investigated by X-ray diffraction (XRD) technique to identify phase formation and crystallinity of the Co-based lattices, and the corresponding patterns are presented in Figure 1a. The XRD spectra of the films exhibit three intense and well-defined diffraction peaks marked with a circle symbol corresponding to the (111), (200), and (220) planes of the nickel foam substrate along with a series of additional diffraction peaks. These substrate reflections are unavoidable due to the high porosity and metallic conductivity of Ni foam, which serves as both the structural backbone and the current collector during the electrodeposition. For the Co_2_CuO_4_ film, the major diffraction peaks appear at 2θ = 31.15°, 36.76°, 38.83°, 59.31°, and 65.19°, which correspond to the (220), (311), (222), (511), and (440) lattice planes of cubic Co_2_CuO_4_ (JCPDS No. 01-1155) [26]. The sharpness and intensity of these reflections confirm the high degree of crystallinity of the electrodeposited film and the successful formation of a single-phase spinel structure. Whereas when the copper precursor was omitted during electrodeposition, the diffraction pattern of the film changed distinctly, producing the typical reflections of cubic Co_3_O_4_ (JCPDS No. 76-1802) [27]. For the Co_3_O_4_ film, the diffraction peaks are located at 2θ = 31.17°, 36.83°, 59.42°, and 65.07°, which can be indexed to the (220), (311), (511), and (440) planes of the spinel Co_3_O_4_ phase. Furthermore, the spinel structure of both Co_2_CuO_4_ and Co_3_O_4_ belongs to the Fd $\bar{3}$ m (227) space group, which offers structural robustness and isotropic electronic pathways. The absence of any extraneous peaks associated with Cu/Co(OH)_2_ or other sub-oxides indicates that Cu is homogeneously incorporated into the cobaltite lattice, confirming the phase purity of the electrodeposited Co_2_CuO_4_ and Co_3_O_4_ films. The diffraction peaks of the Co_2_CuO_4_ are slightly broadened compared to the Co_3_O_4_, which is a result of localized lattice strain arising from the homogeneous substitution of Cu into the Co spinel network. The average crystallite sizes were estimated to be approximately 22.27 nm for Co_2_CuO_4_ and 27.65 nm for Co_3_O_4_ with the help of the (311) diffraction peak using the Debye-Scherrer equation. The slight reduction in crystallite size in Co_2_CuO_4_ indicates that Cu substitution influences nucleation and growth, leading to limited crystal coarsening and subtle structural distortion within the spinel lattice. The structural contrast between Co_2_CuO_4_ and Co_3_O_4_ films demonstrates the vital role of cation composition in dictating the final spinel phase.

**Figure 2:**
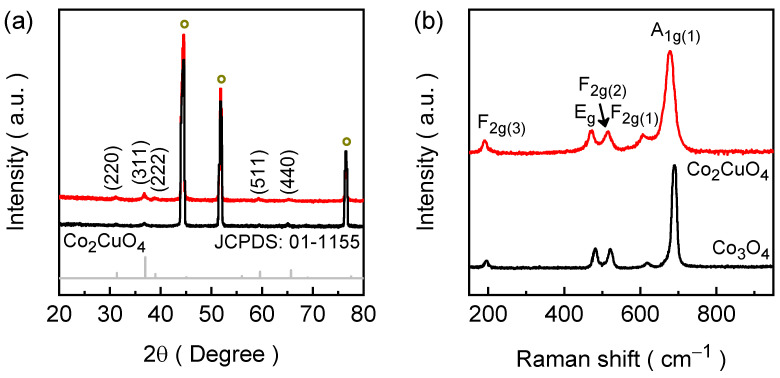
(**a**) XRD spectra along with the standard JCPDS reference patter of Co_2_CuO_4_ and (**b**) Raman spectra of electrodeposited Co_3_O_4_ (black) and Co_2_CuO_4_ (red) films.

Raman spectroscopy was employed to further confirm the crystalline phases and probe structural and vibrational characteristics of the electrodeposited Co_2_CuO_4_ and Co_3_O_4_ films (Figure 2b). The Raman spectrum of Co_2_CuO_4_ displays five characteristic active modes consistent with the cubic spinel structure, including three F_2g_, one E_g_, and one A_1g_ modes [28]. The Raman peaks are centered at 189, 472, 515, 606, and 677 cm^−1^ corresponding to the F_2g(3)_, E_g_, F_2g(2)_, F_2g(1)_, and A_1g(1)_ vibrational modes, respectively [28,29]. The intense A_1g_ mode arises from the symmetric stretching of Co–O bonds involving octahedrally coordinated Co^3+^ cations, while the F_2g_ and E_g_ modes originate from Co^2+^ ions in tetrahedral sites, confirming the dual-valence nature of the spinel framework. The Raman spectrum of the Co_3_O_4_ film also showcases the similar vibrational Raman modes compared to the Co_2_CuO_4_; however, the Raman peaks were slightly blue shifted accompanied by the narrowed Co_2_CuO_4_ Raman bands [30,31]. A comparative broadened band of Co_2_CuO_4_ than Co_3_O_4_ suggest the variation of Co–O bond lengths and symmetry due to the partial substitution of Cu^2+^ within the spinel network, which introduces the local strain, and modifies the electronic environment within the lattice. Both of which are beneficial for improving surface reactivity and charge-transfer efficiency during the catalytic HER. Therefore, together the XRD and the Raman analysis verify that the electrodeposition and subsequent annealing treatments successfully yield phase-pure spinel Co_2_CuO_4_ and Co_3_O_4_ with well-preserved crystallinity.

### 2.3. Chemical Bonding States of Co_2_CuO_4_ than Co_3_O_4_ Films

**Figure 3:**
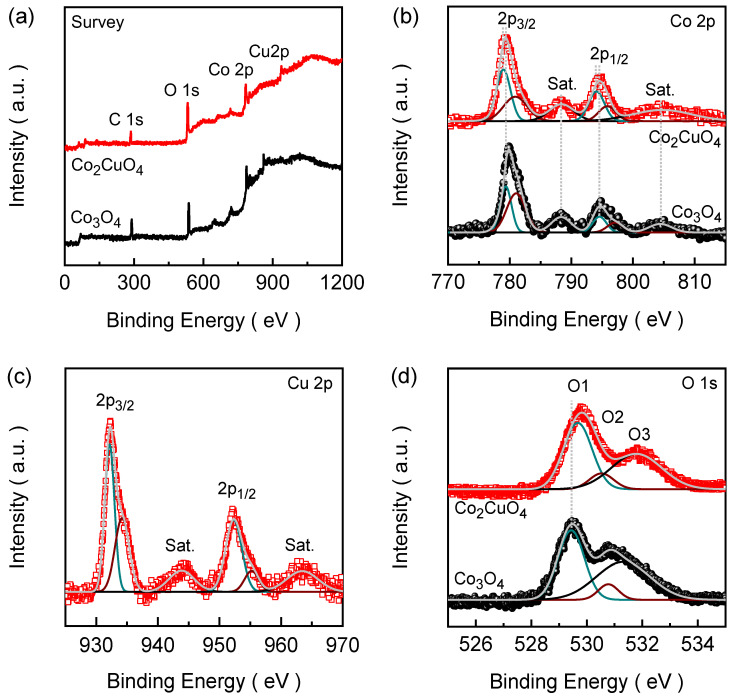
XPS (**a**) Survey spectra; High-resolution (**b**) Co 2p; (**c**) Cu 2p; and (**d**) O 1s emission spectra. All high-resolution spectra were deconvoluted using Gaussian fitting to precisely evaluate the binding energies and corresponding oxidation states.

The survey spectra of Co_2_CuO_4_ and Co_3_O_4_ films (Figure 3a) reveal the presence of Co, Cu, and O without any additional peaks other than C, indicating the phase purity of the spinel oxide. The Gaussian curve fitting model was used to fit the narrow-scan emission spectra of the constituent elements. The Co 2p spectrum of Co_2_CuO_4_ (Figure 3b) shows two main peaks centered at 779.36 eV (Co 2p_3/2_) and 794.87 eV (Co 2p_1/2_) accompanied by characteristic satellite (Sat.) peaks at 788.37 and 804.62 eV [32,33]. After deconvolution, the Co 2p_3/2_ and Co 2p_1/2_ doublets were converted into the spin–orbit components arising from both Co^3+^ (778.98 and 794.16 eV) and Co^2+^ (780.57 and 795.91 eV) species with the spin energy separation of 15.18 eV, confirming the dual-valence Co state consistent with the spinel structure [32,34,35]. In the Cu 2p region (Figure 3c), two major peaks are observed at 932.31 and 952.35 eV, corresponding to Cu 2p_3/2_ and Cu 2p_1/2_ spin–orbit components, respectively, accompanied by two distinct shake-up satellite peaks located at approximately 943.75 and 963.59 eV, which are characteristic of Cu^2+^ species in octahedral coordination [36]. The slight asymmetry and broadening of the Cu 2p_3/2_ peak suggest the coexistence of a minor Cu^+^ component, indicating a mixed-valence Cu^+^/Cu^2+^ configuration that promotes redox reversibility and electronic conductivity within the spinel framework [37]. Figure 3d shows the O 1s spectrum of Co_2_CuO_4_, which was deconvoluted into three distinct components. The O1, O2, and O3 assigned peaks corresponds to the lattice oxygen (OL) at 529.67 eV, oxygen vacancy sites (Ov) at 530.52 eV, and chemisorbed water or oxygen associated with surface hydroxyl species (Oc) at 531.79 eV [38]. The intensity and spin–orbit separation of the Co 2p_3/2_ and Co 2p_1/2_ components along with the O 1s peak feature of pristine Co_3_O_4_ films are almost identical to those of the Co_2_CuO_4_ film. However, compared to the Co_2_CuO_4_, the Co 2p peaks of the Co_3_O_4_ exhibit a slight positive binding energy shift, indicating a redistribution of electron density around Co sites in Co_2_CuO_4_ due to the substitutional incorporation of Cu^2+^ within the spinel lattice [39]. This electronic modification facilitates charge redistribution and strengthens metal-oxygen covalency, ultimately contributing to improved electrocatalytic kinetics.

### 2.4. Electrochemical Properties of Co_2_CuO_4_ than Co_3_O_4_ Electrode Films

**Figure 4:**
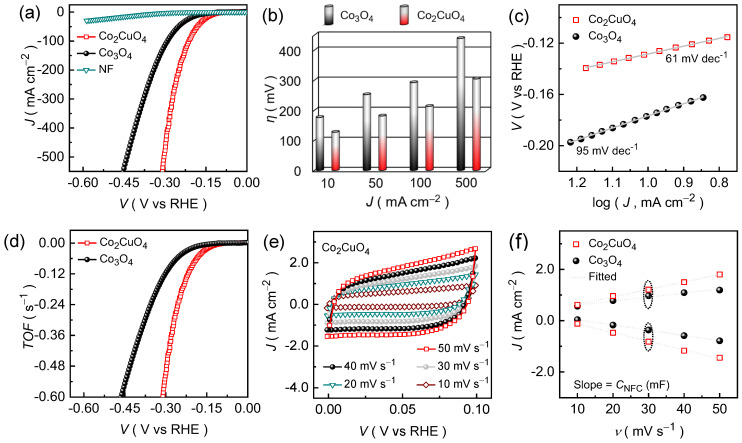
Electrochemical HER activities of Co_2_CuO_4_ and Co_3_O_4_ catalysts evaluated in an alkaline 1.0 M KOH electrolyte medium. (**a**) LSV curves; (**b**) Overpotential as a function of current density; (**c**) Tafel slopes; and (**d**) TOF curves for Co_2_CuO_4_ and Co_3_O_4_ catalysts; (**e**) Non-Faradaic CV curves for Co_2_CuO_4_ and Co_3_O_4_ catalysts; and (**f**) *C*_NFC_ plots for the estimation of ECSA of Co_2_CuO_4_ and Co_3_O_4_ catalysts.

The HER activities of the Co_2_CuO_4_ and Co_3_O_4_ catalysts were assessed using linear sweep voltammetry (LSV) in 1.0 M KOH to elucidate the effect of the binary metal oxide catalysts on catalytic efficiency and durability in an alkaline KOH medium. As shown in Figure 4a, the *iR*-compensated polarization curves reveal a pronounced improvement in activity of the Co_2_CuO_4_ compared with the Co_3_O_4_ catalyst with a lower overpotential and a steeper current response. At a benchmark current density of 10 mA cm^−2^, the Co_2_CuO_4_ catalyst exhibits an overpotential of 127 mV, notably lower than that of pristine Co_3_O_4_ (176 mV). Although both catalysts display similar reaction features, the reduced potential in Co_2_CuO_4_ suggests improved charge-transport efficiency and enhanced accessibility of surface-active sites. The performance distinction between the Co_2_CuO_4_ and Co_3_O_4_ catalysts becomes more evident at higher current densities (Figure 4b), indicating differences in the charge-transfer kinetics and structural stability under the dynamic electrolysis conditions, which correlates well with their morphological features observed in the FE-SEM images (Figure 1). The Co_2_CuO_4_ achieves the current densities of 50, 100, and 500 mA cm^−2^ at an overpotentials of 181, 213, and 304 mV, respectively, while Co_3_O_4_ catalyst requires 252, 292, and 439 mV to drive the same current densities. The smaller potential increment of Co_2_CuO_4_ under dynamic load conditions indicates the better endurance during high-current operation and stronger electrode stability. This enhancement is associated with a comparatively open and interconnected nanosheet framework of Co_2_CuO_4_, which facilitates both electrolyte diffusion and charge transport. The intimate interface between the catalyst layer and the Ni foam substrate further ensures efficient electron/ion migration and mechanical integrity during sustained electrolysis.

To elucidate the underlying HER kinetics, the Tafel slope analysis was performed using the polarization curves. Figure 4c shows the obtained Tafel curves of the Co_2_CuO_4_ and Co_3_O_4_ catalysts. The measured Tafel slopes are 61 mV dec^−1^ for the Co_2_CuO_4_ catalyst and 95 mV dec^−1^ for Co_3_O_4_ catalyst, confirming the faster charge-transfer kinetics in the former Co_2_CuO_4_ catalyst. In alkaline media, the HER typically proceeds through a two-step sequence involving the adsorption of hydrogen intermediates followed by their subsequent desorption as molecular hydrogen from the catalyst surface. The reaction initiates with the Volmer step (theoretical Tafel slope = 120 mV dec^−1^), wherein water molecules are reduced at the active metal sites (M) to form hydroxide ions (OH^−^) and adsorbed hydrogen species (MH_ads_):

M + H_2_O + e^−^ → MH_ads_ + OH^−^, (3)

The subsequent hydrogen evolution occurs through the electrochemical desorption process (Heyrovsky reaction, theoretical Tafel slope = 40 mV dec^−1^):

MH_ads_ + H_2_O + e^−^ → M + OH^−^ + H_2_, (4)

or through the chemical recombination (Tafel reaction, theoretical Tafel slope = 30 mV dec^−1^) step:

2 MH_ads_ → 2 M + H_2_, (5)

The experimentally measured Tafel slopes (61 and 95 mV dec^−1^) lie between the theoretical limits of the Volmer (120 mV dec^−1^) and Heyrovsky (40 mV dec^−1^) processes, suggesting that the HER kinetics on both catalysts predominantly follow the Volmer-Heyrovsky mechanism, where water dissociation and electrochemical desorption occurs sequentially [40]. The smaller Tafel slope of Co_2_CuO_4_ signifies a faster charge-transfer rate and a lower kinetic barrier for hydrogen release, implying that the electrochemical desorption (Heyrovsky step) acts as the rate-determining step. These kinetic trends are consistent with the LSV results and demonstrate the strong correlation between catalytic structure and HER activity, which is further supported by turnover frequency (TOF) analysis.

The intrinsic reaction kinetics of the Co_2_CuO_4_ and Co_3_O_4_ catalysts were further evaluated by estimating the TOF values, which reflect the activity of individual catalytic sites. The TOF values were calculated using the relation [41]:

*TOF* = (*J* · *A*)/(*n* · *N* · *F*), (6)

where *J* represents the current density (A cm^−2^), *A* denotes the geometric area of the electrode (1 × 1 cm^2^), *F* signifies the Faraday constant (96,485 C mol^−1^), *n* designates the number of electrons transferred per hydrogen molecule (*n* = 2 for HER), and *N* corresponds to the number of moles of active catalyst loaded on the electrode. As shown in Figure 4d, the “*V* vs. TOF” plots clearly demonstrate that Co_2_CuO_4_ catalyst exhibits significantly higher TOF values across the entire potential range compared with the Co_3_O_4_ catalyst, indicating superior intrinsic activity per active site. The TOF values at an overpotential of 300 mV for Co_2_CuO_4_ was calculated to be approximately 0.504 s^−1^, which is nearly five-fold higher than that of pure Co_3_O_4_ (0.116 s^−1^) at the same potential. Moreover, the Co_2_CuO_4_ catalyst achieves a mass activity of 0.641 A mg^−1^, which is nearly 3.8-fold higher compared to the Co_3_O_4_ (0.169 A mg^−1^) catalyst at the same reference potential. This pronounced enhancement highlights the accelerated catalytic turnover capability of Co_2_CuO_4_, reflecting its improved charge-transport efficiency and higher density of accessible active sites, which are strongly linked with the open nanosheet architecture. The interconnected nanosheet framework offers numerous catalytically exposed sites and shorter ion diffusion pathways, while ensuring efficient electron transport through the continuous conductive network (Figure S2a and Table S2).

Thereafter, the electrochemically active surface area of the Co_2_CuO_4_ and Co_3_O_4_ catalysts was estimated from the non-faradaic capacitance (*C*_NFC_) values, obtained from cyclic voltammetry (CV, Figure 4e and Figure S2b) curves recorded at various scan rates within the non-faradaic potential window using the following relation:

*ECSA* = *C_NFC_*/*C_E_*, (7)

*C_NFC_* = *J*_NFC_/*v*, (8)

where *J*_NFC_, *v*, *C_E_* represents the non-faradaic current density, scan rate, and KOH electrolyte capacitance [42]. The anodic and cathodic current densities were plotted as a function of scan rate (Figure 4f) and the slope of the resulting linear fit correspond to *C*_NFC_. The Co_2_CuO_4_ catalyst exhibits a considerably steeper slope compared to the Co_3_O_4_ catalyst, demonstrating the higher *C*_NFC_ value of 31.33 mF cm^−2^ compared to the Co_3_O_4_ (~18.17 mF cm^−2^). Since *ECSA* is directly proportional to the non-faradaic capacitance, the larger *C*_NFC_ of Co_2_CuO_4_ indicates a greater density of electrochemically accessible sites for catalytic HERs, attributed to its highly interconnected nanosheet architecture, which provides abundant active edges and open channels that promote rapid electrolyte infiltration and charge exchange. The higher *ECSA* of the Co_2_CuO_4_ catalyst facilitates the enhanced water molecule adsorption and the efficient charge accumulation at the electrode-electrolyte interface, thereby accelerating HER kinetics.

**Figure 5:**
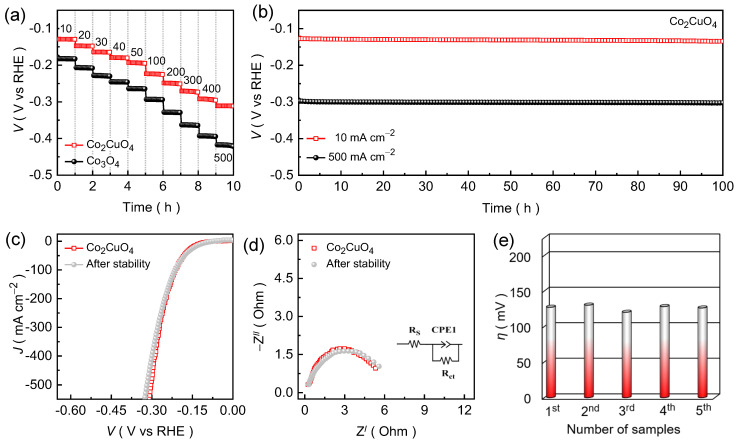
(**a**) Voltage step profile being function of scan rate for the Co_2_CuO_4_ and Co_3_O_4_ catalysts evaluated in an alkaline 1.0 M KOH electrolyte medium; (**b**) Chronopotentiometric stability curves recorded over 100 h at 10 and 500 mA cm^−2^; Post stability measured (**c**) LSV and (**d**) EIS curves for the Co_2_CuO_4_ catalyst; (**e**) Reliability of the HER performance for Co_2_CuO_4_ catalysts evaluated through series of catalyst testing.

Chronopotentiometric curves were recorded as a function of current density for the Co_2_CuO_4_ and Co_3_O_4_ catalysts (Figure 5a) to further assess their catalytic robustness and dynamic stability under an alkaline KOH medium. The applied cathodic current density was systematically increased from 10 to 50 mA cm^−2^ with a step of 10 mA cm^−2^ and subsequently raised in steps of 100 mA cm^−2^ up to 500 mA cm^−2^. Interestingly, both catalysts display steady potential response during continuous operation, demonstrating excellent stability under varying current loads. The Co_2_CuO_4_ catalyst consistently sustains lower potential values throughout the entire current range than the Co_3_O_4_ catalysts, confirming the enhanced catalytic efficiency and superior endurance even at a high-current HER evaluation. The nearly static voltage response at each step without noticeable degradation highlights the strong interfacial adhesion between the catalyst layer and the Ni foam substrate, ensuring mechanical and electrochemical stability under vigorous hydrogen gas evolution conditions. The linear relationship between potential and current density further confirms efficient charge-transfer during the stepped electrolysis, indicating high electrode reliability and effective ionic transport within the porous 2D nanosheet structure. Furthermore, the long-term durability of Co_2_CuO_4_ catalyst was then evaluated through extended chronopotentiometric testing at fixed current densities of 10 and 500 mA cm^−2^ for a continuous duration of 100 h (Figure 5b). The Co_2_CuO_4_ catalyst maintains a remarkably stable potential response throughout the entire stability test without significant alteration in the potential value. The sustained voltage stability of Co_2_CuO_4_ catalyst demonstrated outstanding structural integrity and robust charge-transport pathways during the extended hydrogen evolution. The post-stability LSV curves (Figure 5c) and EIS spectra (Figure 5d) exhibit nearly identical polarization profiles and minimal variation in the charge-transfer resistance for the Co_2_CuO_4_ catalyst relative to the initial measurement, verifying that the catalyst retains its intrinsic activity and electrode integrity after prolonged HER operation. Nonetheless, the reliability test (Figure 5e and Figure S3) was conducted using five independently fabricated Co_2_CuO_4_ and Co_3_O_4_ catalyst, which reveals an almost identical electrochemical responses, indicating the excellent reproducibility of the catalytic performance under alkaline HER conditions.

## 3. Materials and Methods

### 3.1. Materials

Analytical-grade reagents purchased from Sigma-Aldrich (St. Louis, MO, USA) were used in all experiments without any further purification. Cobalt(II) chloride hexahydrate (CoCl_2_·6H_2_O, ≥98%), copper(II) chloride dihydrate (CuCl_2_·2H_2_O, ≥98%), ammonium hydroxide solution (NH_4_OH, ≥99.99%), and potassium hydroxide (KOH, ≥85%) were used for material fabrication and testing. Hydrochloric acid (HCl, 37%), ethanol (CH_3_CH_2_OH, ≥95%), and Acetone (CH_3_COCH_3_, ≥99.5%) were employed as cleaning and processing agents. Three macroporous nickel foam used as the conductive substrate for electrode preparation. Before electrodeposition, the Ni foam pieces were sequentially ultrasonicated in ethanol, diluted HCl, deionized water, and acetone to eliminate surface contaminants and ensure good adhesion of the deposited films.

### 3.2. Synthesis of Co_3_O_4_ and Co_2_CuO_4_ Electrodes

The Co_2_CuO_4_ nanosheet film was synthesized on Ni foam through a facile electrodeposition technique. In a typical procedure, an aqueous electrolyte bath 50 mL was prepared in a glass beaker using CuCl_2_·2H_2_O and CoCl_2_·6H_2_O under continuous stirring at room temperature, and a pre-cleaned Ni foam was subjected to an electrodeposition using VersaSTAT instrument (Ametek Scientific Instruments, Berwyn, PA, USA). The Ni foam, Pt wire, and saturated calomel electrode (SCE) served as the working, counter, and reference electrode, respectively. The electrodeposition process was performed for 300 s at a biasing potential of −1.0 V (vs. SCE). The (Co-Cu) hydroxide precursor film was dried overnight followed by air annealing for 2 h at 350 °C to obtain the desired Co_2_CuO_4_ film. For comparison, the Co_3_O_4_ film was also synthesized using a similar electrodeposition process, except the utilization of CuCl_2_·2H_2_O in the electrolyte solution bath. The experimental conditions were optimized based on the preliminary experimental results. The detailed FE-SEM description is presented in the Supporting Information section (Figure S4).

### 3.3. Material Characterization

The structural, morphological, and chemical characteristics of the prepared Co_3_O_4_ and Co_2_CuO_4_ were comprehensively analyzed using various advanced techniques. The crystal structure and phase purity were examined by high-resolution X-ray diffraction (XRD) using Cu Kα radiation (λ = 1.5406 Å). The XRD spectra were recorded at spectral angle ranging between 20° and 80°. The surface chemical composition and oxidation states of the constituent elements were investigated using X-ray photoelectron spectroscopy (XPS) on a Phi 5000 VersaProbe Scanning Microprobe (ULVAC-Phi, Chigasaki, Japan). All binding energies were calibrated using the carbon C 1s peak situated at 286.53 eV as a reference. The surface morphology and microstructural features were observed using field-emission scanning electron microscopy (FE-SEM) and the elemental composition and spatial distribution were determined through energy-dispersive X-ray spectroscopy (EDX) integrated into the SEM system. Raman spectroscopy was performed on a LabRam Aramis spectrometer (Horiba Jobin Yvon, Yongin, Republic of Korea) using a 514 nm Ar-ion laser as the excitation source to analyze the vibrational characteristics and structural features of the film samples.

### 3.4. Electrochemical Test

The hydrogen evolution reaction (HER) characteristics of the fabricated Co_2_CuO_4_ and Co_3_O_4_ electrodes were assessed on a VersaSTAT electrochemical workstation using a conventional three-electrode setup with 1.0 M KOH as the alkaline electrolyte. The catalyst-coated nickel foam served as the working electrode, while a graphite rod and a SCE served as the counter and reference electrodes, respectively. All electrochemical tests were conducted at 25 ± 2 °C in freshly prepared 1.0 M KOH solution purged with high-purity argon for at least 30 min prior to measurement to remove dissolved oxygen. Linear sweep voltammetry (LSV) experiments were performed in the potential window range between 0.0 to −1.6 V (vs. SCE) at a scan rate of 1.0 mV s^−1^ to determine the polarization behavior. The measured SCE potential scale (i.e., *E*_SCE_) was changed to the reversible hydrogen electrode (RHE) scale (i.e., *E*_RHE_) and the internal ohmic losses were normalized using *iR*-compensation (90%), where the series resistance (*Rs*) was obtained from the high-frequency intercept of the Nyquist plot in EIS measurements, as shown in the following equations [43]:

*E*_RHE_ = *E*_SCE_ + (0.059 × *pH*) + *E*_SCE_°, (9)

*η* = *E*_RHE_ − (*J* × *R*s), (10)

where *pH* and *E*_SCE_° are the hydrogen-ion concentration in the electrolyte solution and the standard potential of SCE at room temperature. The reaction kinetics were then analyzed by plotting Tafel curves, which were derived from the linear portion of the polarization data and fitted according to the following equation [6]:

*η* = *a* + (*b* × log(*J*)), (11)

where *a* and *b* are the arbitrary constant and Tafel slope, respectively. The stability and durability of the electrode were examined through chronopotentiometric testing at constant current density over extended operation periods. The accessible electrochemically active surface area (*ECSA*) for the Co_3_O_4_ and Co_2_CuO_4_ electrodes was estimated through non-Faradaic cyclic voltammetry (CV) curves. The non-Faradaic CV curves were recorded within the potential range between 0.00 and 0.10 V (vs. RHE) at various scan rates and the non-Faradaic capacitance (*C*_NFC_) was estimated to approximate the density of accessible active sites. The electrochemical impedance spectroscopy (EIS) measurements were conducted at a fixed overpotential with a 10 mV AC amplitude between the frequency range of 0.01 and 10 kHz to probe the charge-transfer resistance (*Rct*).

## 4. Conclusions

In summary, a systematic comparison of Co_2_CuO_4_ and Co_3_O_4_ nanosheet catalysts demonstrates how cationic substitution and electronic modulation significantly influence hydrogen evolution behavior under alkaline conditions. The incorporation of Cu^2+^ into the Co_3_O_4_ spinel framework tailors the electronic structure, enhances conductivity, and introduces a mixed-valence configuration of copper and cobalt, all of which collectively promote faster reaction kinetics and efficient charge-transportation. The Co_2_CuO_4_ catalyst exhibits notably lower overpotential of 127 mV, smaller Tafel slopes of 61 mV dec^−1^, and higher TOF values than pristine Co_3_O_4_ catalyst, corroborating its superior intrinsic catalytic activity. The enlarged electrochemically active surface area and reduced interfacial resistance further ensure rapid charge-transfer and efficient mass transport. The long-term chronopotentiometric stability tests up to 100 h at 10 mA cm^−2^ validate its mechanical robustness and excellent durability, maintaining steady performance even under high current density of 500 mA cm^−2^. Importantly, this work provides mechanistic insight into how Cu incorporation modulates the Co–O bonding environment and electronic configuration within the spinel lattice, resulting in enhanced orbital hybridization between Cu 3d, Co 3d, and O 2p states. This electronic reconstruction effectively lowers the energy barrier for intermediate adsorption and accelerates the Volmer-Heyrovsky process during HER. Overall, these findings establish a rational Cu-substitution strategy that couples structural and electronic tuning, offering a deeper understanding of activity enhancement mechanisms and a scalable pathway toward high-efficiency transition-metal oxide catalysts for sustainable hydrogen production.

## Data Availability

The original contributions presented in this study are included in the article/Supplementary Material. Further inquiries can be directed to the corresponding author.

## Supplementary Materials

The supporting information can be downloaded at: https://www.mdpi.com/article/10.3390/ijms262211226/s1. References [44,45,46,47,48,49,50,51] were cited in Supplementary Materials.

## Abbreviations

The following abbreviations are used in this manuscript:

HER	Hydrogen evolution reaction
Pt	Platinum
FE-SEM	Field-emission scanning electron microscopy
EDX	Energy-dispersive X-ray spectroscopy
XRD	X-ray diffraction
XPS	X-ray photoelectron spectroscopy
SCE	Saturated calomel electrode
RHE	Reversible hydrogen electrode
EIS	Electrochemical impedance spectroscopy
*J*_NFC_	Non-faradaic current density
*C*_NFC_	Non-faradaic capacitance
*ECSA*	electrochemically active surface area
CV	Cyclic voltammetry
LSV	Linear sweep voltammetry
TOF	Turnover frequency
*η*	Overpotential
Rct	Charge-transfer resistance
*F*	Faraday’s constant
*A*	Geometric area of the electrode
*N*	Number of moles of active catalyst
*n*	Number of electrons transferred per hydrogen molecule
