# Supplementary material for: Unlocking the Cu-Co Interplay: Electrodeposited Spinel Co2CuO4 as a High-Performance Hydrogen Evolution Catalyst
Source: Int J Mol Sci. 2025 Nov 20;26(22):11226. doi: 10.3390/ijms262211226 (PMC12653121; doi:10.3390/ijms262211226)
Supplement: Supplementary file 1 [file ijms-26-11226-s001.zip › ijms-3988030-supplementary.pdf]

Supporting Information

Unlocking the Cu-Co Interplay: Electrodeposited Spinel Co_2CuO_4 as a High-Performance Hydrogen Evolution Catalyst

Sankar Sekar [1,2], Momin M. Mujtaba [3], Abu Saad Ansari [4], Sangeun Cho [1], Youngmin Lee [1,2], Sejoon Lee [1,2] and Abu Talha Aqueel Ahmed [1,*]

[1] Division of System Semiconductor Science, Dongguk University, Seoul 04620, Republic of Korea

[2] Quantum-functional Semiconductor Research Center, Dongguk University-Seoul, Seoul 04620, Republic of Korea

[3] M.S.G. Arts, Science, & Commerce College, Malegaon-camp, 423203, India

[4] Nano Center Indonesia Research Institute, Puspiptek Street, South Tangerang, Banten 15314, Indonesia

Corresponding Author: abutalha.aa@dongguk.edu

S1. Supporting Materials and Methods

S1.1. Synthesis of Co_3O_4 electrodes

To optimize the deposition duration, Co_3O_4 films were initially grown at different times (150, 300, and 450 s). SEM analysis (Figure S1) revealed that short deposition (150 s) produced sparse and discontinuous nanosheets, while prolonged deposition (450 s) resulted in disproportionate stacking. In contrast, the film obtained at 300 s exhibited a well-defined, vertically aligned nanosheet network with open interstitial spaces, favoring electrolyte penetration and efficient ion diffusion.

Table S1. Comparative electrocatalytic HER performance of the optimized Co_2CuO_4 catalyst with various binary metal-based catalysts in alkaline 1.0 M KOH electrolyte at a current density of 10 mA cm^{-2}.

No.	Catalyst film	Overpotential @10 (mA cm^{-2})	Tafel slope (mV dec^{-1})	Stability at *J* (*J* in mA cm^{-2})	Supporting Reference
1	Co_2FeO_4@PdO	269	49	48 h@20	[S1]
2	Co_2FeO_4	372	103	-	
3	$CuCo_2O_4$	115	153	30 h@10	[S2]
4	P-doped $CuCo_2O_4$	152	115.8	15 h@-0.15 V	[S3]
5	Co_3S_4@MoS_2	280	74	10 h@1.51 V	[S4]
6	$NiCo_2Se_4$/$NiCoS_4$	180	107.4	12 h@-1.3 V	[S5]
7	$NiCo_2Se_4$	207	151.7	-	
8	$CuCo_2O_4$/CoOOH/NF	125	75.1	15 h	[S6]
9	$CuCo_2O_4$/NF	223	204.5	-	
10	$CuCo_2O_4$	168	113	-	[S7]
11	$MnCo_2S_4$	111	63	50 h@500	[S8]
12	$MnCo_2O_4$	233	128	-	
**13**	**Co_2CuO_4**	**127 mV**	**61**	**100 hrs.@10**<br>**100 hrs.@500**	**Present work**

Supporting Figures

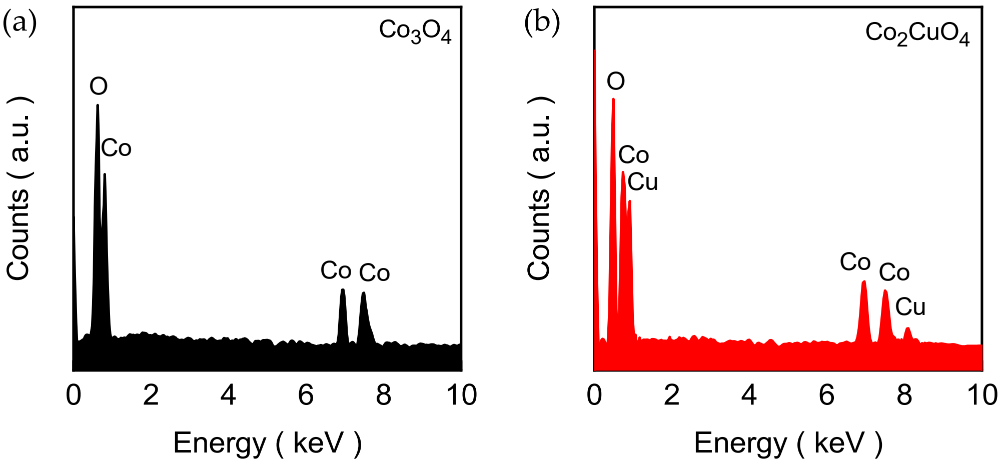

Figure S1. EDX spectra of (**a**) Co_2CuO_4 and (**c**) Co_3O_4 catalyst electrodes. The inset tables display the elemental distribution, outlining the atomic percentage ratios of the respective elements.

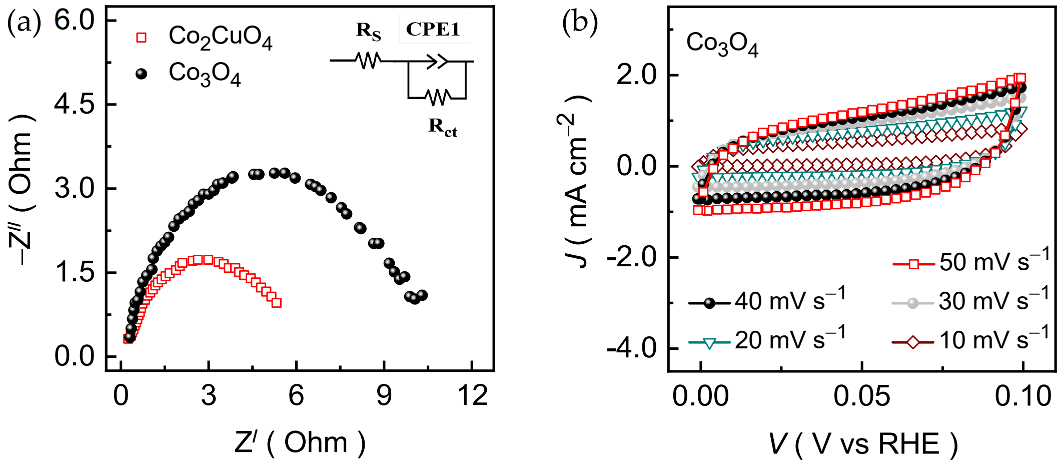

Figure S2. (**a**) EIS curves of Co_2CuO_4 and Co_3O_4 catalysts along with the tank circuit used to fit the Nyquist curves and (**b**) Non-Faradic CV curves recorded at various scan rates for Co_3O_4 catalyst.

Further insight into the interfacial charge dynamics was provided by electrochemical impedance spectroscopy (EIS). The Nyquist plots (Figure S2a) display a single semicircular feature. The high-frequency intercept with the real axis in the EIS curve represents the solution resistance (*Rs*). Whereas the semicircle diameter indicative of charge-transfer resistance (*Rct*). All fitted impedance parameters values obtained from equivalent circuit modeling are summarized in Table S2. The Co_2CuO_4 catalyst possesses a much smaller *Rs* and *Rct* than Co_3O_4 catalyst, demonstrating its superior electronic conductivity and faster charge-transfer dynamics. This enhancement can be attributed to Cu incorporation into the Co_3O_4 lattice, which promotes the charge delocalization and reduces interfacial resistance. The improved conductivity correlates well with the lower Tafel slope and higher turnover frequency, confirming the synergistic effect of Cu doping on the overall HER kinetics.

Table S2. Nyquist impedance data fitted using Z-view software for the Co_2CuO_4 and Co_3O_4 catalysts.

Catalysts	Before stability				After HER stability			
	R_S (Ω)	CPE1-T	CPE1-P	R_{ct} (Ω)	R_S (Ω)	CPE1-T	CPE1-P	R_{ct} (Ω)
**Co_2CuO_4**	0.149	0.000335	0.75	5.56	0.149	0.000287	0.73	5.61
**Co_3O_4**	0.155	0.000129	0.78	9.91	-	-	-	-

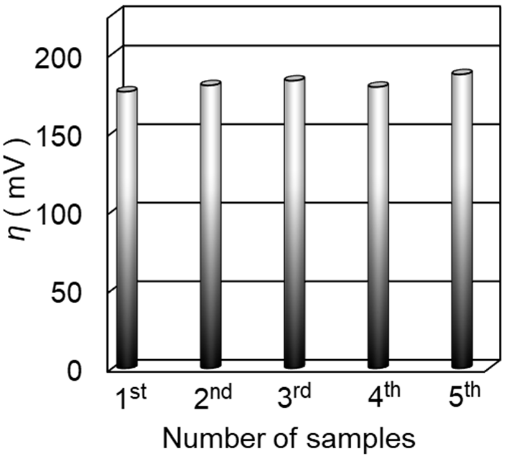

Figure S3. Reliability data of Co_3O_4 catalysts examined using the series of catalyst electrodes obtained at the same experimental conditions.

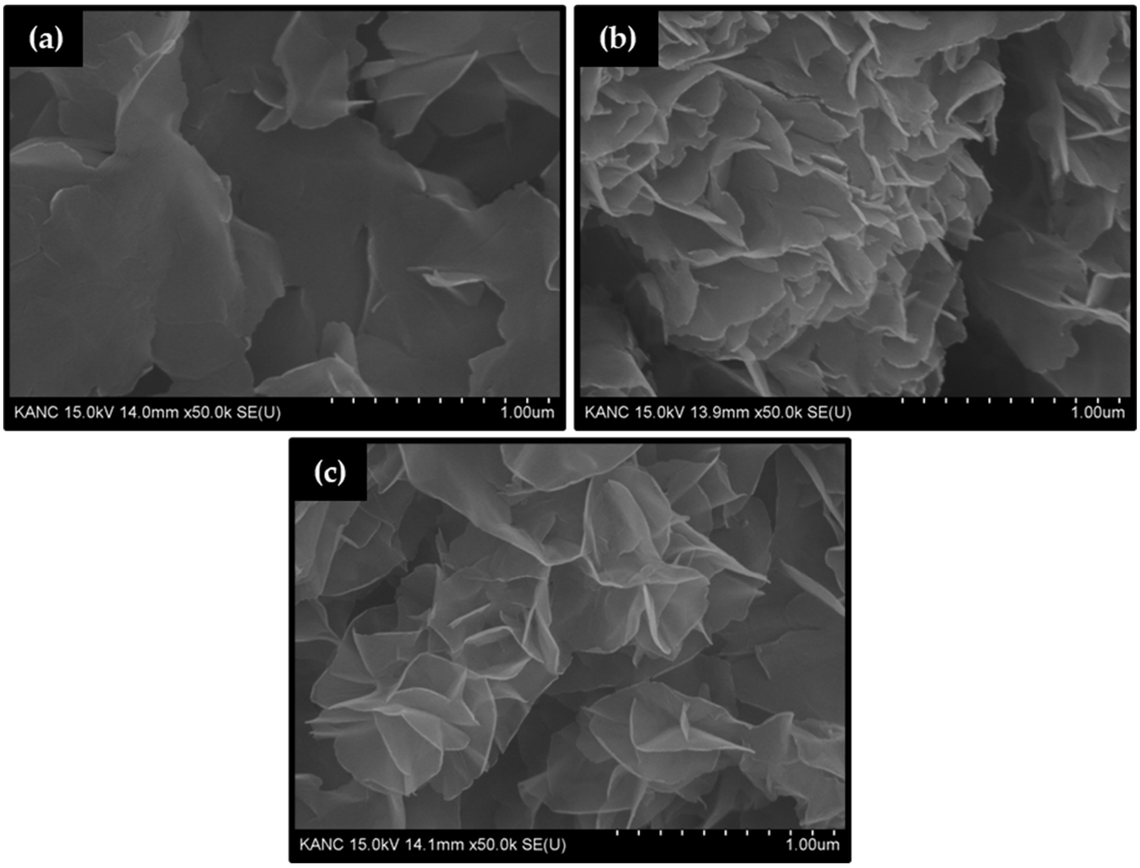

Figure S4. FE-SEM images of Co_3O_4 catalyst grown for (**a**) 150 s, (**b**) 300 s, and (**c**) 450 s during electrodeposition.

Supporting References

1. Hanan, A.; Lakhan, M. N.; Shu, D.; Hussain, A.; Ahmed, M.; Soomro, I. A.; Kumar, V.; Cao, D., An efficient and durable bifunctional electrocatalyst based on PdO and Co_2FeO_4 for HER and OER. *Int. J. Hydrogen Energy* **2023,** *48* (51), 19494-19508.
2. Aqueel Ahmed, A. T.; Pawar, S. M.; Inamdar, A. I.; Kim, H.; Im, H., A Morphologically Engineered Robust Bifunctional $CuCo_2O_4$ Nanosheet Catalyst for Highly Efficient Overall Water Splitting. *Adv. Mater. Interfaces* **2020,** *7* (2), 1901515.
3. Tan, S.; Ji, Y.; Ren, F.; Chen, F.; Ouyang, W., Improved energy conversion and storage performance enabled by hierarchical zigzag-like P-doped $CuCo_2O_4$ nanosheets based 3D electrode materials. *Int. J. Hydrogen Energy* **2022,** *47* (15), 9248-9260.
4. Guo, Y.; Tang, J.; Wang, Z.; Kang, Y.-M.; Bando, Y.; Yamauchi, Y., Elaborately assembled core-shell structured metal sulfides as a bifunctional catalyst for highly efficient electrochemical overall water splitting. *Nano Energy* **2018,** *47*, 494-502.
5. Wang, K.; Lin, Z.; Tang, Y.; Tang, Z.; Tao, C.-L.; Qin, D.-D.; Tian, Y., Selenide/sulfide heterostructured $NiCo_2Se_4$/$NiCoS_4$ for oxygen evolution reaction, hydrogen evolution reaction, water splitting and Zn-air batteries. *Electrochim. Acta* **2021,** *368*, 137584.
6. Wang, C.; Jiu, H.; Zhang, L.; Song, W.; Zhang, Y.; Wei, H.; Xu, Q.; Che, S.; Guo, Z.; Qin, Y., Bifunctional $CuCo_2O_4$/CoOOH as a synergistic catalyst supported on nickel foam for alkaline overall water splitting. *J. Alloys Compd.* **2022,** *929*, 167367.
7. Zequine, C.; Wang, F.; Li, X.; Guragain, D.; Mishra, S. R.; Siam, K.; Kahol, P. K.; Gupta, R. K., Nanosheets of $CuCo_2O_4$ As a High-Performance Electrocatalyst in Urea Oxidation. *Applied Sciences* **2019,** *9* (4), 793.
8. Ahmed, A. T. A.; Sekar, S.; Khadtare, S. S.; Rochman, N. T.; Chinna, B.; Ansari, A. S., Anion-exchange synthesis of an $MnCo_2S_4$ electrocatalyst towards facilitated ultralong hydrogen evolution reaction in acidic and alkaline media. *CrystEngComm* **2024,** *26* (2), 215-222.
